# Editorial: Lifestyle and environmental factors and human fertility

**DOI:** 10.3389/fendo.2025.1771853

**Published:** 2026-01-30

**Authors:** María Laura Ribeiro

**Affiliations:** Laboratorio de Fisiología y Farmacología de la Reproducción, Centro de Estudios Farmacológicos y Botánicos (CEFYBO), Facultad de Medicina (National Research Council (CONICET) - University of Buenos Aires (UBA)), Buenos Aires, Argentina

**Keywords:** environmental exposures, fertility, in vitro fertilization, lifestyle, nutrition, predictive models, socio-environment

## Introduction

Human fertility is a complex and multifactorial process influenced by genetic, hormonal, physiological, behavioral, and environmental determinants. A growing body of evidence has highlighted a concerning global trend: declining fertility rates and an increasing prevalence of reproductive disorders in both men and women.

Fertility rates have declined worldwide over the past several decades, with most countries now below the replacement level of 2.1 children per woman. This trend is especially pronounced in industrialized and high-income regions, but is increasingly observed globally (1–7). Recent estimates show over 55 million men and 110 million women affected globally, with the burden increasing steadily from 1990 to 2021 and projected to continue rising through 2040 (5–9). Male reproductive health is deteriorating, evidenced by declining sperm counts, increased testicular cancer, and more reproductive system abnormalities (4, 8, 10). Female infertility is also increasing, with the highest burden among women aged 35-39, and a trend toward earlier onset (1, 9, 11). High-income countries show declining fertility and, in some cases, a decrease in primary infertility, but secondary infertility and reproductive disorders remain concerns (1, 2, 7). Low- and middle-income regions are experiencing a rapid rise in infertility, often linked to infections, limited healthcare access, and environmental exposures (5, 6, 8, 9). Aging populations and delayed parenthood further exacerbate infertility risks, especially for women (1, 9, 11).

While part of this decline can be attributed to socio-demographic and cultural changes -such as delayed parenthood, economic pressures, and access to contraception- there is mounting concern that modifiable factors related to lifestyle and environmental exposures play a crucial role in shaping reproductive health outcomes. Lifestyle factors including diet, physical activity, alcohol consumption, tobacco use, stress, and sleep quality are now recognized as key modulators of reproductive function (12–17).

At the same time, environmental influences -ranging from air pollution and industrial contaminants to occupational exposures and climate change- have become increasingly relevant in the context of fertility (18–20). Pollutants such as heavy metals, pesticides, and microplastics can interfere with hormone signaling, oxidative stress pathways, and DNA integrity, ultimately affecting both gamete quality and embryonic development.

Importantly, the impact of these factors may extend across generations. There is strong evidence -especially from animal models- that environmental and lifestyle factors can induce epigenetic changes in the germline, which are transmitted across generations and increase reproductive risk (21, 22). While human data are less direct, the potential for transgenerational inheritance of reproductive disorders is a growing concern, warranting further research and preventive action. Given this complex interplay between biological systems, lifestyle behaviors, and environmental conditions, interdisciplinary research has become essential. The integration of epidemiological data, clinical observations, molecular insights, and experimental models would allow for a comprehensive understanding of how modern living environments influence human reproductive capacity.

## Insights from this Research Topic

The Frontiers in Endocrinology Research Topic “Lifestyle and Environmental Factors and Human Fertility” brings together contemporary research addressing this multifaceted challenge. The Topic provides a platform that collectively advances our understanding of the intricate relationships between lifestyle, environment, and reproductive health with the goal of fostering a multidisciplinary dialogue among scientists and clinicians investigating the diverse determinants of human reproductive health.

**Figure 1:**
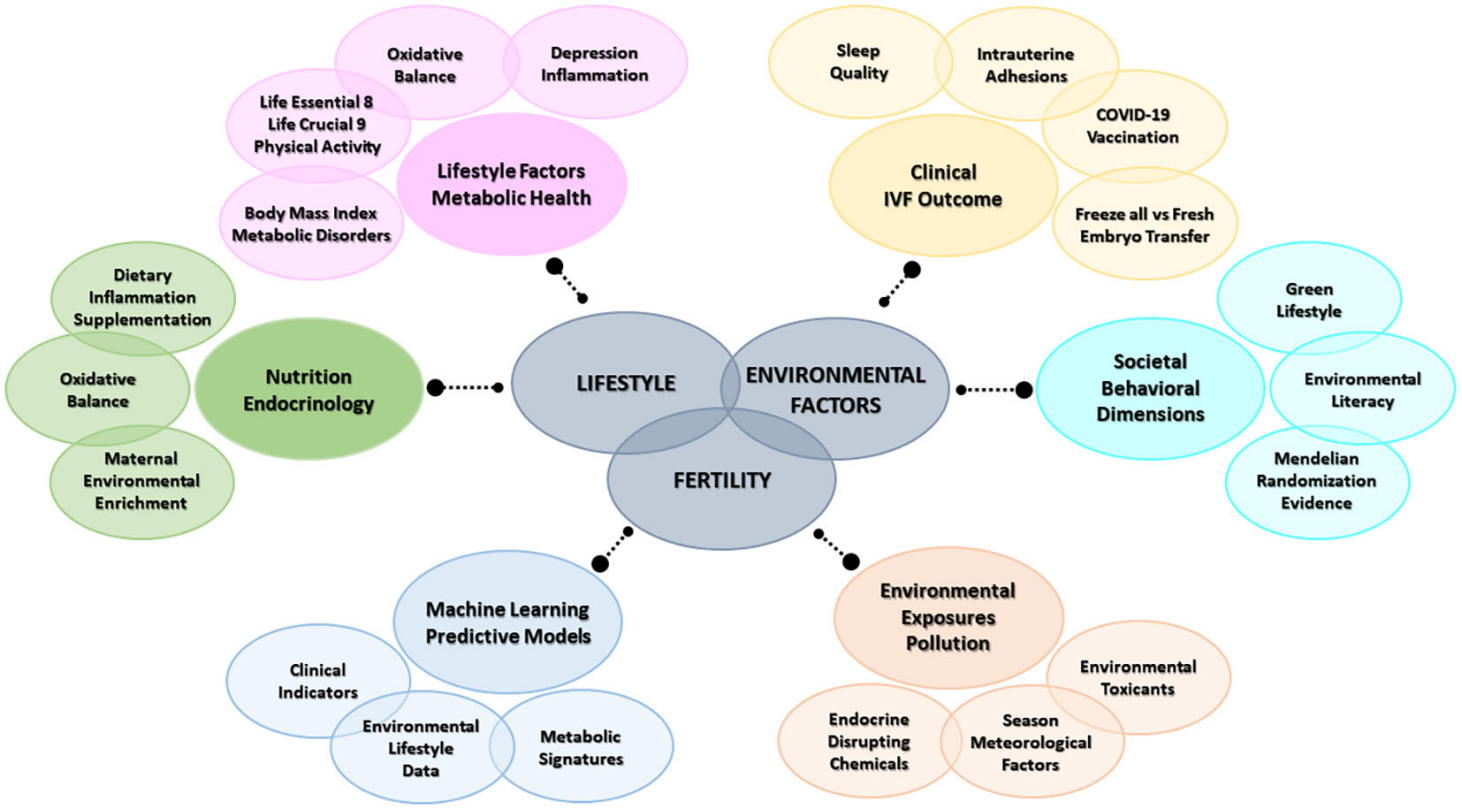
Topic integrative perspectives.

Recognizing that fertility decline cannot be fully explained by demographic or genetic factors alone, the Topic sought to integrate perspectives from: 1) lifestyle factors and metabolic health, 2) environmental exposures and pollution, 3) machine learning, omics and predictive models, 4) nutrition, diet and endocrine regulation, 5) clinical and reproductive outcomes, and 6) socio-environmental and behavioral dimensions (**Figure 1**). By doing so, it aimed to capture the multifactorial nature of reproductive impairment and to identify actionable strategies for prevention and intervention. Collectively, these contributions explore the effects of dietary habits, physical activity, body composition, environmental pollutants, occupational exposures, psychosocial stressors, and circadian rhythm disturbances on human fertility (**Table 1**). Across diverse methodologies -from machine learning prediction models and large-scale epidemiological analyses to experimental and clinical investigations- these studies reveal converging evidence that metabolic health, oxidative balance, environmental toxicants, and psychosocial well-being are deeply intertwined with fertility outcomes in both women and men. Research exploring the Life’s Essential 8 and Life’s Crucial 9 indices (Gu et al., Cui et al., Huang et al., Zhang et al., Pang et al.) highlights the value of integrative clinical metrics in predicting infertility risk, while investigations into arsenic exposure (Su et al.), air pollution (Chen et al.), pesticides (Liu et al.), and endocrine disruptors (Tricotteaux-Zarqaoui et al., Sciorio et al.) reinforce the reproductive consequences of environmental contaminants. Studies addressing sleep quality, body composition, and inflammatory dietary patterns (Xie et al., Liu et al., Liang et al., Xia et al., Ding et al.) further emphasize the complex behavioral and metabolic mediators of reproductive potential. Complementary research on assisted reproductive technologies, vaccination, and seasonality (Wei et al., Ouyang et al., Li et al., Han et al., Shui et al., Li et al.) reflects how clinical and environmental variables jointly shape reproductive outcomes. Experimental findings from animal models (De la Cruz Borthiry et al.) and nutritional and physical interventions (Najdgholami et al., Chen et al.) provide mechanistic insights into hormonal regulation and reproductive resilience. Taken together, this Research Topic advances a holistic understanding of fertility as a reflection of both biological integrity and environmental stewardship, calling for interdisciplinary approaches that integrate public health, environmental science, and reproductive medicine.

**Table 1:** Topic contributions.

	Authors	Study Type/Data Source	Main Findings
Lifestyle Factors and Metabolic Health	Gu X et al.	Machine learning model (NHANES, SHAP method)	Combined Life´s Essential 8 (LE8) and heavy metal exposure predict female infertility risk
	Cui S et al.	Observational and mediation analyses (LE8)	Depression, inflammation and metabolic disorders mediates LE8´s effect on infertility
	Liu M et al.	Cross-sectional (NHANES)	Body roundness index independently linked to females infertility
	Huang S et al.	Epidemiology study	Life´s Crucial 9 (an expanded LE8) protects against infertility among U.S. women
	Xia P et al.	Population-based study	
	Chen P et al.	Logistic regression and mediation analyses (NHANES)	Physical activity mitigates infertility risk via lower HbA1c
Environmental Exposures and Pollution	Su L et al.	Cross-sectional (NHANES)	Urinary arsenic positively associated with endometriosis prevalence
	Chen M et al.	Retrospective IVF cohort	Maternal PM2.5 exposure linked to preterm birth and miscarriage in IVF patients
	Liu F et al.	Popultaion-based cross-sectional	Maternal pesticide exposure increases risk of birth defects
	Li H et al.	Cohort (IVF outcomes)	Season and meteorological factors influence embryo transfer outcomes
	Sciorio R et al.	Narrative review	Environmental toxicants impair sperm quality and fertility potential
	Tricotteaux-Zarqaoui S et al.	Review	Endocrine-disrupting chemicals (EDCs) alter fertility via epigenetic mechanims
Machine Learning, Omics and Predictive Models	Gu X et al.	ML model (SHAP)	Combined environmental and lifestyle data yield robust infertility prediction
	Zhang R et al.	Machine learning model	Clinical indicators accurately predict infertility and pregnancy loss
	Ding N et al.	Metabolomics profiling	Distinct metabolic signatures identify women at risk of pregnancy loss
Nutrition, Diet and Endocrine Regulation	Liang M et al.	Mediation analyses (NHANES)	Dietary inflammatory and oxidative balance scores predict reproductive function
	Najdgholami Z et al.	RCT (PCOS)	Flaxseed supplementation improves reproductive endocrine profiles
	De la Cruz Borthiry F et al.	Environmental enrichment	Maternal environmental enrichment increases corpora lutea and progesterone levels
Clinical and Reproductive Outcomes	Xie Y et al.	Prospective birth cohort	Sleep quality differs between ART and naturally conceiving women
	Ouyang et al.	IVF cohort study	Intrauterine adhesions impair endometrial receptivity and IVF outcomes
	Wei J et al.	Cohort	COVID-19 vaccination does not negatively affect IVF outcomes
	Han Y et al.	Systematic review and meta-analyses	Freeze-all vs fresh embryo transfer outcomes in adenomyosis and endometriosis
Socio-environmental and Behavioral Dimensions	Li Y et al.	Survey study	Identified determinants promoting green lifestyles among Chinese residents
	Shui Y et al.	Cross-sectional (rural Sichuan)	Environmental literacy cprrelates with better health outcomes
	Pang Q et al.	Review of Mendelian randomization studies	Summarized MR-evidence on common male-specific diseases affecting fertility

Importantly, the Topic sought to underscore the interconnected nature of these influences. Rather than acting independently, lifestyle and environmental exposures often converge to amplify reproductive risk. This integrative approach reflects a paradigm shift in reproductive endocrinology: from studying isolated risk factors toward a systems-level understanding of fertility as an indicator of overall health. The assembled articles collectively reinforce the idea that human fertility can serve as a sensitive biomarker of broader physiological and ecological well-being.

In summary, this Research Topic represents a comprehensive, evidence-based synthesis of how modern living conditions affect reproductive potential. By bringing together observational, mechanistic, and clinical insights, it lays the groundwork for future translational research aimed at mitigating reproductive risks through lifestyle modification, environmental policy, and public health action. The 24 articles published in this Research Topic collectively address how lifestyle patterns and environmental conditions interact to modulate human reproductive capacity. Across the categories investigated, a unifying message emerges: fertility reflects systemic health. Whether the trigger is metabolic imbalance, environmental pollution, or psychosocial strain, the reproductive axis responds as a sensitive indicator of physiological stability. The evidence gathered collectively supports the notion that reproductive endocrinology is inseparable from broader lifestyle and environmental sciences.

## Scientific and societal relevance

The cumulative findings of this Research Topic highlight the profound intersection between human reproductive health and broader societal and environmental contexts. Fertility is not merely a clinical endpoint; it is a sensitive biomarker reflecting systemic physiological integrity, environmental exposures, and lifestyle patterns. Understanding these interconnections carries both scientific significance and societal urgency.

From a scientific perspective, the articles collectively advance knowledge in several domains. First, they provide mechanistic evidence linking metabolic and nutritional factors to gamete quality and reproductive outcomes. This reinforces the concept that metabolic health is a modifiable determinant of fertility, opening opportunities for targeted interventions. Second, research on environmental pollutants underscores the vulnerability of the reproductive system to even low-level exposures, highlighting the need for stringent monitoring and regulatory policies. Third, studies addressing psychosocial stress, circadian disruption, and physical activity emphasize that lifestyle behaviors operate through complex endocrine and epigenetic pathways, offering additional avenues for preventive strategies.

The societal relevance of these findings is equally compelling. Globally, fertility rates are declining in many regions, and the burden of infertility is increasingly recognized as both a public health challenge and a social concern (4). Its consequences extend far beyond individual health to impact families, communities, and national development. By identifying modifiable lifestyle and environmental risk factors, the Research Topic contributes actionable knowledge that can inform health promotion, reproductive counseling, and population-level interventions. For instance, promoting balanced diets, regular yet moderate physical activity, and adequate sleep, alongside policies to reduce exposure to air pollution, could enhance fertility outcomes and improve overall health.

Moreover, these studies illuminate the interdisciplinary nature of reproductive science. Fertility research no longer resides solely within endocrinology or gynecology; it spans toxicology, epidemiology, nutrition, behavioral science, and environmental policy. This integrated perspective enriches scientific understanding and strengthens the evidence base for translational and preventive strategies that can benefit individuals and populations alike.

Finally, the Research Topic underscores the importance of equity and access. Environmental exposures and lifestyle-related risks are often unevenly distributed across socio-economic strata. Recognizing and addressing these disparities is essential for achieving reproductive justice, ensuring that all individuals have the opportunity to maintain reproductive health regardless of geographic, economic, or social constraints. The 24 articles collectively demonstrate that human fertility is both a marker of personal health and a reflection of societal and environmental conditions. They provide a compelling case for integrating lifestyle optimization and environmental stewardship into public health agendas, while simultaneously expanding the scientific horizons of reproductive endocrinology.

## Future directions and concluding remarks

The research compiled in this Research Topic provides a comprehensive snapshot of how lifestyle and environmental factors influence human fertility, yet it also highlights the complexity and remaining gaps in our understanding. Despite substantial progress, many mechanisms linking metabolic status, environmental exposures, and psychosocial stress to reproductive function remain incompletely elucidated. For instance, the interplay between multiple exposures -metabolic, chemical, and behavioral- requires integrative models capable of capturing synergistic or antagonistic effects. Future studies employing systems biology approaches, longitudinal cohorts, and advanced biomarker analyses will be critical to untangle these relationships.

Another promising avenue lies in translational and preventive research. While clinical studies increasingly incorporate lifestyle counseling, robust evidence-based guidelines are still limited. Interventions targeting diet, physical activity, sleep, and environmental risk reduction could be standardized and tested across diverse populations. Similarly, elucidating the molecular signatures of environmental and lifestyle exposures may allow the development of early biomarkers of reproductive risk, enabling timely intervention before fertility is compromised.

From a societal perspective, research must continue to address health disparities and environmental justice. Many populations disproportionately experience high environmental exposures or limited access to reproductive care, emphasizing the need for inclusive studies and policies that prioritize vulnerable groups. Global collaboration, open data sharing, and interdisciplinary partnerships will be essential to translate scientific insights into equitable solutions.

For young investigators entering this field, the Research Topic offers both inspiration and guidance. Fertility research is no longer confined to isolated laboratories or clinical silos; it intersects endocrinology, toxicology, epidemiology, nutrition, behavioral science, and public health. Embracing this interdisciplinary mindset offers opportunities to make meaningful contributions that resonate far beyond the lab, influencing policy, health outcomes, and societal well-being.

In conclusion, lifestyle and environmental factors are powerful determinants of human reproductive health, reflecting both individual choices and broader ecological conditions. The studies presented here demonstrate that interventions at multiple levels -molecular, clinical, behavioral, and societal- have the potential to preserve and enhance fertility. As the field moves forward, a combination of rigorous science, innovative methodologies, and a commitment to public health and equity will be crucial. By integrating these perspectives, the next generation of researchers can not only deepen scientific understanding but also foster a future in which reproductive health is safeguarded as a vital component of human well-being.
